# Lipidure-based micropattern fabrication for stereotyping cell geometry

**DOI:** 10.1038/s41598-023-47516-8

**Published:** 2023-11-22

**Authors:** Drew B. Grespin, Talen G. Niven, Riley O. Babson, Erich J. Kushner

**Affiliations:** Department of Biological Sciences, University of Denver, Denver, CO 80210 USA

**Keywords:** Angiogenesis, Actin, Cell culture, Tissue culture

## Abstract

Cell autonomous behaviors such as migration and orchestration of cell polarity programs are required for physiological tissue formation. Micropatterns are cell-adhesive shapes that confine cell(s) to a user defined geometry. This biophysical confinement allows researchers to standardize the cell shape, and in doing so, stereotype organelle and cytoskeletal systems that can have an arbitrary organization. Thus, micropatterning can be a powerful tool in interrogation of polarity programs by enforcing a homogenous cell shape and cytoskeletal organization. A major drawback of this approach is the equipment and reagent costs associated with fabrication. Here, we provide a characterization of a compound called Lipidure (2-Methacryloyloxy ethyl phosphorylcholine) that is up to 40X less expensive than other cell repulsive coating agents. We found that Lipidure is an effective cell-repulsive agent for photolithography-based micropattern fabrication. Our results demonstrate that Lipidure is sensitive to deep UV irradiation for photolithography masking, stable in both benchtop and aqueous environments, non-toxic in prolonged culture, and effective at constraining cell geometry for quantification of cytoskeletal systems.

## Introduction

An inherent issue with interpreting and quantifying cytoskeletal behaviors in cells is that many of the outputs are temporally transient and spatially stochastic in 2-dimensional cell culture. For instance, isolated cells maintained on a plastic, or glass coverslips are highly migratory but typically lack directionality, randomly change course, and are generally considered nonpolarized^1^. In this example, attempting to assign a leading edge, or a cell front with high confidence can be challenging and somewhat arbitrary. Additionally, in vivo, cell migration does not typically involve an empty, planar surface. Instead, all cell types are spatially confined by neighboring tissue structures which can aid in tissue guidance. A promising compromise is to geometrically constrain cell(s) to a user defined shape using micropatterns^2^. This will maintain all the necessary imaging advantages of 2D culture to resolve dynamic structures, while dramatically reducing the inherent stochastic morphology of the cell shape, cytoskeleton, etc. This, in turn, significantly lowers the biological variability, enhancing one’s ability to quantify differences in intrinsically dynamics systems. Generally, this tool is underutilized in cell biology and could significantly aid in elucidating aspects of cytoskeletal programs.

Micropattern construction combines a cell repulsive layer coupled with a cell adhesive coating in a desired shape^2^. There are many methods for achieving this end-product and innumerable geometries that could be tailored to almost any given biological question^3^. One common method employed to fabricate micropatterns is photolithography^4^. Photolithography harnesses deep UV (< 200 nm) light to excite a photo-sensitive compound, through a photomask. The photomask itself normally made of chromium and quartz will harbor transparent openings of the user-defined shapes allowing for UV light to pass through for the selective destruction of the underlying cell repulsive layer creating a void. Thereafter, voided areas can be backfilled with a cell-adhesive extracellular matrix (ECM) of choice. This photomasking technique has several advantages over other methods such as contact-micropatterning using ECM-loaded stamps or plasma cleaning^5,6^, namely due to its reproducibility and improved feature resolution. Drawbacks to this methodology include the initial equipment investment and the consumable cost, which can be prohibitive for some laboratories. Currently, most techniques also suffer from a relatively short shelf-life, forcing users to fabricate and use micropatterns in a compressed timeline. While micropatterns are commercially available, they are engineered for a limited number of use cases and cell types. Additionally, commercial micropatterns are not an economically viable option if numerous micropatterns are needed due to the relatively high cost per unit.

Herein, we demonstrate a use case for Lipidure® (Lipidure, 2-Methacryloyloxy ethyl phosphorylcholine co-polymer) as a low-cost, effective, and long-lived cell repulsive agent for photolithographic micropattern fabrication. As previously reported^7^, Lipidure can be coated directly onto glass coverslips or grafted onto polystyrene for enhanced ECM adherence and is a suitable compound for photolithography. Our characterization suggests that Lipidure can be obtained in gram quantities, is stable on a benchtop for up to 90 days and maintains its cell-repulsiveness in liquid culture for a week or more. Lipidure-coated surfaces can be heat-sterilized without functional consequences. Lastly, we show the effectiveness of Lipidure-based micropatterns for enforcing defined cell polarities. Overall, we present a method for deep UV based micropatterning that can be applied to probing aspects of cytoskeletal biology.

## Results

### Lipidure is compatible with deep UV-based micropatterning

**Figure 1:**
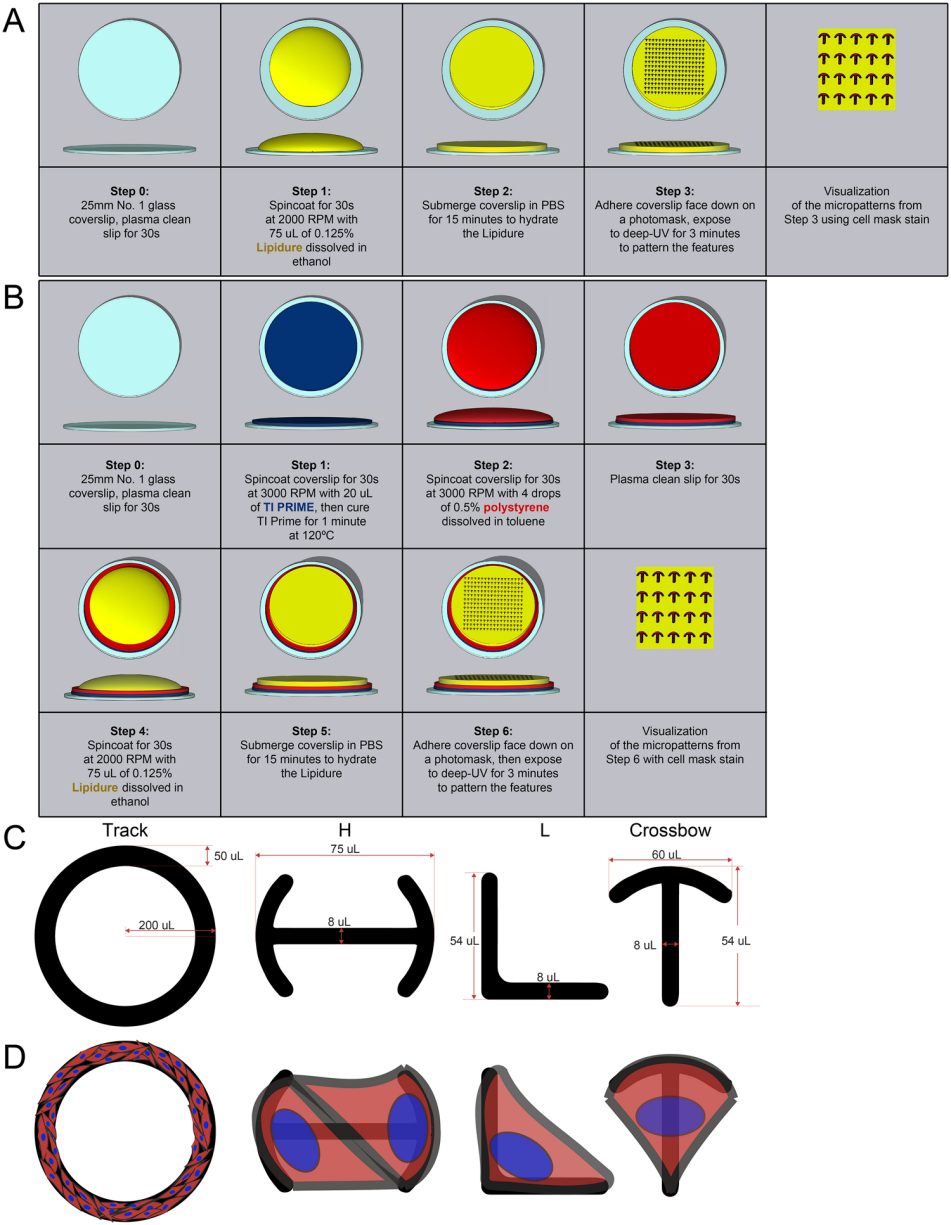
Overview of micropatterning surface preparation and masking protocol. (**A**) Schematic of adhering Lipidure to a glass coverslip and UV-based micropatterning. (**B**) Schematic of adhering polystyrene and Lipidure to a glass coverslip. Coated coverslips were subjected to masking and UV exposure to create micropattern features. (**C**) Diagram of micropattern features with dimensions in µm. (**D**) Predicted cell orientation on indicated micropatterns.

Lipidure is a polymer originally developed for surface preparation of artificial organs^8^. Each monomer harbors several hydrophobic phosphorylcholine moieties rendering coated surfaces highly resistant to protein and cell attachment. Lipidure can be stored at room temperature in atmospheric conditions and obtained in gram quantities at a reasonable cost. For these qualities, we first tested if Lipidure would be a suitable cell repulsive agent for photolithographic micropatterning. Using a standard coverslip preparation protocol^4^, we coated glass coverslips with 0.125% Lipidure either on glass or polystyrene-coated coverslips (Fig. 1A,B). An initial concern was the extent to which Lipidure was sensitive to deep UV exposure to void the features for cells to adhere. To explore this, we fabricated a photomask with 4 different shapes optimized for an endothelial cellular footprint (Fig. 1C) but could also be applied to other tissue systems. First, we fabricated a larger track approximately the width of a capillary (50 µm). Thereafter, we devised a ‘H’ pattern to hold two cells creating a common junction, a ’crossbow’ pattern to enforce a forward cell polarity in single cells, and a ‘L’ pattern to create a long actin filament in single cells. These features were based off previous investigations shown to enforce the aforementioned cell behaviors^5,9^. Between the large and smaller patterns, we believed this provided an ample assessment of Lipidure’s sensitivity to deep UV exposure, resulting feature resolution and compatibility with cell culture.

**Figure 2:**
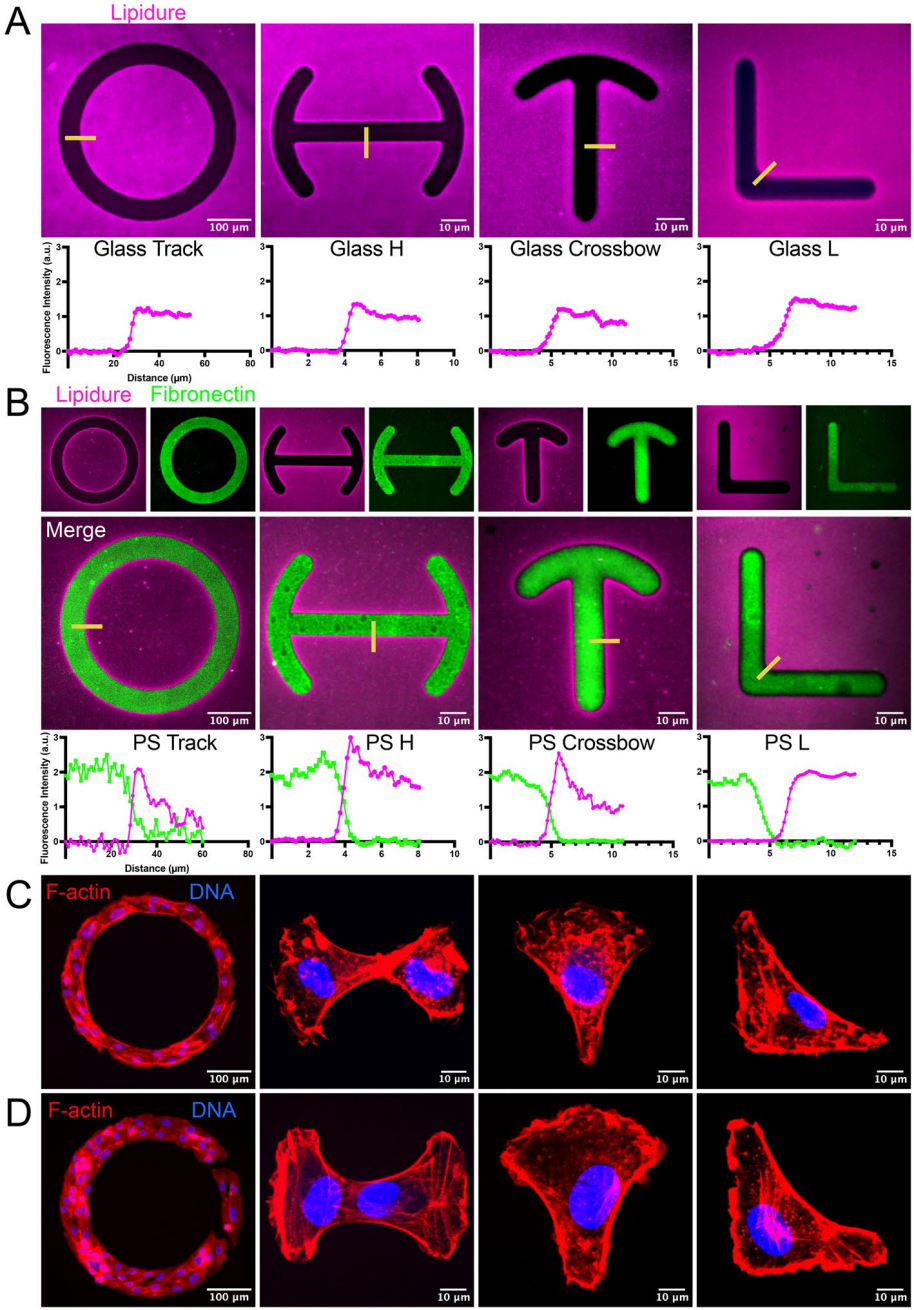
Lipidure is sensitive to deep-UV exposure and capable of micropatterning endothelial cells. (**A**) Representative images of coverslip coated with 0.125% Lipidure after masking/ultraviolet (UV) exposure and then stained with Cell Mask™ dye to highlight the UV-voided areas. Line scans correspond to yellow lines in top panels. (**B**) Representative images of coverslips coated with polystyrene and then coated with 0.125% Lipidure. After masking and UV exposure, fluorescent-dye conjugated fibronectin was grafted and then Lipidure was stained with Cell Mask dye. Line scan graphs correspond to yellow lines in top panels. (**C**) Human umbilical vein endothelial cells (ECs) adhered to glass-only micropatterns, stained for actin and DNA as indicated. (**D**) ECs adhered to polystyrene and fibronectin-coated micropatterns. ECs were stained for actin and DNA as indicated.

We observed that spin-coating 0.125% Lipidure provided a continuous, transparent surface (Supplementary Fig. 1B,C). Another helpful feature of Lipidure chemistry is that cell membrane dyes can be used to mark Lipidure in minutes allowing for rapid visualization of coating and patterning quality. Next, we assessed if the Lipidure coating could be selectively destroyed via photomasking and deep UV exposure. Both on glass and polystyrene, deep UV effectively voided the underlying Lipidure layer creating the intended micropattern feature (Fig. 2A,B). As assessed by line scan analysis, in all patterns we observed a sharp border between the Lipidure and feature, indicating a high-level of resolution (Fig. 2A,B). This data suggests that Lipidure can be destroyed by a deep UV source and is suitable for masking-based fabrication as previously reported^7^.

A polystyrene coating allows for ECM proteins of choice to backfill the patterned areas. Unlike glass (borosilicate), polystyrene polymers are highly degraded by short UV exposure rendering the surface reactive to substrate grafting. Relying on this trait, after masking we incubated a polystyrene-coated coverslip with fluorescently conjugated fibronectin. As predicted, the fibronectin efficiently grafted into the patterned shape with no detectable attachment to the surrounding Lipidure-coated area (Fig. 2B). Line scan analysis demonstrated a sharp border between the cell repulsive Lipidure and fibronectin features. These results suggest that ECM grafting is achievable using Lipidure.

Our next goal was to ensure that cells would adhere to the micropatterns and stay within the confines of the features on both glass and polystyrene. To test this, we plated endothelial cells (ECs) on both glass and fibronectin covered micropatterns. ECs that were applied to glass only micropatterns required 2–3 h to fully adhere, while ECs added to fibronectin micropatterns typically adhered in 15–20 min. On both surfaces, ECs conformed to the expected shapes and did not show evidence of significantly breaching the Lipidure interface (Fig. 2C,D). We also tested adherence of HEK-293 and normal human lung fibroblasts cells. Both cell types demonstrated excellent cell adhesion and growth on the Lipidure voided substrates (Supplementary Fig. 1A). Overall, these results demonstrate Lipidure is an effective cell-repulsive agent that can be utilized in micropattern fabrication.

### Lipidure stability

**Figure 3:**
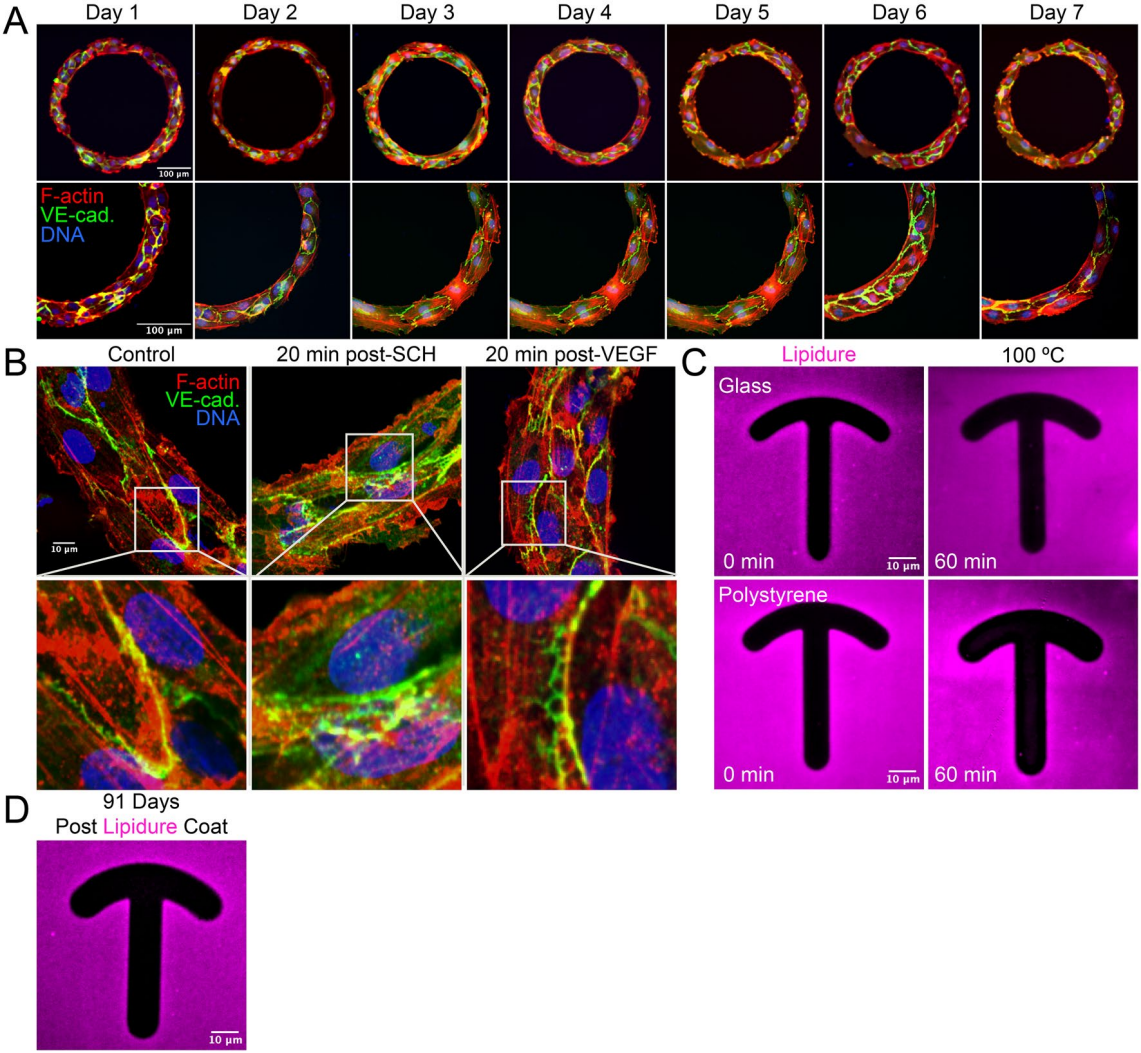
Lipidure is stable in prolong cell culture. (**A**) Representative images of Human umbilical vein endothelial cells (ECs) plated on ‘track’ micropatterns cultured between 1 and 7 days. Bottom panels are areas of higher magnification. ECs were stained as indicated in bottom panels. (**B**) Representative images of ‘track’ micropatterns treated with DMSO (control), ERK inhibitor SCH772984 (10 μM) or vascular-endothelial growth factor (VEGF, 20 ng/μl) 165 ligand for 20 min (min). Thereafter, ECs were stained for indicated proteins. Bottom panels indicate areas of higher magnification.

We next sought to test several parameters of Lipidure stability both in, and out of, cell culture. First, we seeded ECs on a glass track pattern and cultured for 7 days to determine if there was any potential toxicity of Lipidure as well as its ability to continuously constrain cells in an aqueous environment. Our results demonstrate that prolonged culture is feasible with Lipidure as we did not see any evidence of cellular distress (e.g. excessive membrane blebbing, or lack of proliferation). Additionally, there were no signs of Lipidure degradation as ECs were maintained within the confines of the track pattern over the duration of the experiment (Fig. 3A). As a potential use case, we next stained for the junctional protein VE-cadherin. A common assay in angiogenic-related research is visualizing junctional proteins for insights into vascular stability. A general issue with this approach is culturing a monolayer in which the cellular density and number of cell–cell contacts are homogenous. The use of a track pattern allowed for rapid seeding and saturation of the adherent areas to produce a homogenous monolayer. To test for differences in junctional stability, we incubated patterned cells with SCH772984, an ERK inhibitor, or vascular endothelial growth factor-A ligand (VEGF-A). As previously published^10,11^, both agents promoted a more serrated VE-cadherin appearance in line with a destabilized phenotype (Fig. 3B). These data suggest Lipidure is useful for extended EC culture experiments.

We next tested how Lipidure reacted to prolonged heat exposure. The reasoning for this was that all steps of fabrication were performed in a non-sterile environment. Thus, our question was could we heat-sterilize the micropatterns prior to culture? To answer this, we placed the micropatterns in a 100 °C oven for varying amounts of time, then, after fluorescently tagging the Lipidure, we imaged the micropattern features to probe for any signs of heat-related degradation. We observed that pattern integrity was not affected by prolonged heat exposure (Fig. 3C). We were also interested in how long Lipidure was stable in atmospheric conditions at room temperature. After 91 days of bench top storage, we did not detect any appreciable degradation in Lipidure as assessed by staining (Fig. 3D). It is likely that Lipidure can be stored for more than 3 months; however, this was not tested. Overall, our data suggests that Lipidure micropatterns are extremely stable in culture, sterilizing-heat, and in long-term dry storage conditions.

### Employing Lipidure-based micropatterns in stereotyping cytoskeletal systems in ECs

**Figure 4:**
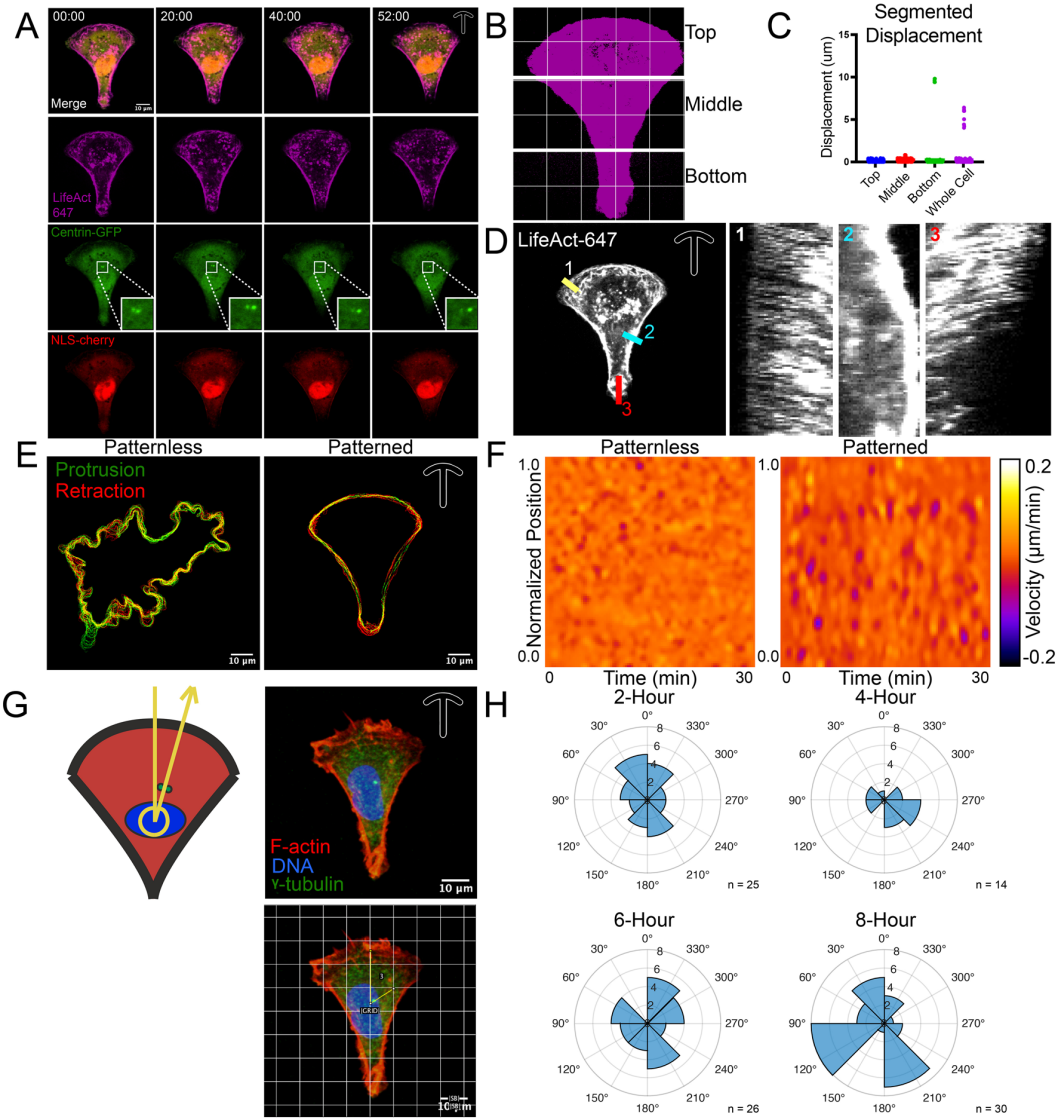
Using Lipidure-based micropatterns to stereotype endothelial cell geometry. (**A**) Representative live-cell imaging of a Human umbilical vein endothelial cell (EC) plated on a ‘crossbow’ micropattern expressing LifeAct-TagRFP647, Centrin-GFP, and NLSmCherry. (**B**) Binary image of micropatterned cell trisected into labeled regions. (**C**) Quantification of membrane displacement of trisected regions noted in panel B or whole cell analysis. (**D**) Representative cell and associated kymographs of actin (LifeAct) dynamics over time. (**E**) Non-micropatterned (patternless) and micropatterned EC with protrusive (green) and retractive (red) cell membrane movements marked over time as analyzed by APAPT software. (**F**) Heatmap derived from cells in panel E for visualization of cell membrane velocity over time. (**G**) Diagram and example of nuclear-centrosome axis angle quantification. ECs on crossbow micropatterns were aligned with the bow portion up and a central vertical line in the middle of the cell. Nuclear-centrosome axis was determined by drawing a line originating from the centroid of the nucleus passing through the centrosome cluster. (**H**) Compass plots of nuclear-centrosome axis over various plating times.

Micropatterning forces cells that would be adopting a somewhat random shape to conform to precise geometric arrangements. In turn, cytoskeletal systems also assume a stereotyped organization that would otherwise be more stochastic compared with a freely migrating cell. This control of cell shape and associated structural organization greatly enhances an investigator’s ability to detect cytoskeletal or planar cell polarity differences between experimental groups by markedly decreasing variability associated with shape differences. To demonstrate this utility, we adhered ECs on fibronectin-coated crossbow micropatterns while expressing LifeAct-TagRFP647, Centrin-GFP, and NLS-mCherry (Fig. 4A). To glean an understanding of how stable the actin cytoskeleton was in this configuration, we divided each cell into the following regions: top, middle, and bottom (Fig. 4B). We also determined the mean displacement of the cell edges using ADAPT^12^ in the aforementioned regions as well as for the entire cell and observed a low level of membrane movement in the micropattern EC (Fig. 4B–D). Next, we compared a micropatterned cell to a freely migrating EC. Again, using ADAPT, we marked the cells protrusive and retractive movements at the plasma membrane periphery. There was a stark reduction in both membrane protrusions and retractions as compared to an unconstrained cell (Fig. 4E,F). Overall, these data demonstrate Lipidure-based micropatterns can be used in conjunction with live-imaging techniques for quantification of cellular dynamics.

We next used the crossbow micropatterns to quantify the nuclear-centrosome axis as a marker of planar cell polarity as previously reported^9^. Indeed, stereotyping the cellular geometry allowed us to accurately plot the nuclear centrosome directionality overtime (Fig. 4F–H). We observed that the nuclear-centrosome axis trended towards the anterior bow over time. We contend that this is useful as freely migrating cells have no inherent directionality; thus, this method can be used in determining potentially minute perturbations in polarity signaling otherwise undetectable in non-micropatterned cells.

## Discussion

Herein, we present a simple variation of a micropatterning technique using Lipidure, a new, low-cost, cell repulsive agent. We show that Lipidure is sensitive to deep UV irradiation, thus making it a suitable substrate for photolithography. Lipidure-based micropatterns are capable of enforcing cell geometry and are compatible with long-term culture. We also establish that Lipidure-based micropatterns are useful in creating uniform cell geometries for better quantification of cytoskeletal systems. In cell biological research, this is a generally underutilized approach to understand cell autonomous programs and behaviors. In this realm, micropatterning could be used to further probe aspects of cell competition, junctional stability, polarity shifts in response to stimuli, etc.

In theory, any cell-repulsive coating can be used to constrain cells in a user defined shape; however, finding a cell-repulsive agent that is non-toxic, stable, UV sensitive and low cost is rare. Our laboratory is not the first to use Lipidure as a cell repulsive agent as others have also demonstrated this compounds suitability for micropattern fabrication^6,7^. However, to date, we believe our work is the first to test Lipidure’s general stability and suitability for long-term culture experiments. As compared with microcontact printing or an aluminum foil mask using plasma, photomasking allows for higher resolution of patterned features. Microcontact-based micropattern fabrication can suffer from low reproducibility as compared to methods that do not require the use of stamps to impart adhesive features. There are other techniques that may be comparable to the resolution and reproducibility of Lipidure-based micropatterns such as using gold nanodots^13^; however, the authors have not performed a direct comparison.

The most prominent micropatterning coating are variants of PLL(20)-g[3.5]-PEG(2): poly-L-lysine-g-poly (ethylene glycol) (PEG). Although, this a very effective coating agent, it costs up to $12,000USD/gram on the high end and 3,100USD/gram on the low end as compared to Lipidure at $300USD/gram. On a per coverslip basis, Lipidure and PEG (assuming the lowest price) are virtually equivalent in cost (see Supplementary Information). With the highest price of PEG, Lipidure can be a 40X reduction in cost. In this report, we did not perform a head-to-head comparison to PEG as Lipidure was more than adequate on all tested micropatterning parameters. Overall, these techniques, equipment, and reagents are becoming less expensive, side-stepping the need for cleanroom facilities to engineer micropatterns. In turn, this affords biological researchers the ability to create their own unique patterns that best suit their experimental question(s) and/or model of interest.

## Materials and methods

### Data availability and reagents

Data, analytic methods, and study materials will be made available to other researchers upon written request to the corresponding author. All research complied with the University of Denver Institutional Biosafety Committee (IBC). All reagents and plasmid information are listed in the reagents table in the Supplementary Information (Supplementary Tables 1, 2, 3 and 4).

### Cell culture

Pooled Human umbilical vein ECs (HUVECs, PromoCell) were cultured in proprietary media (PromoCell Growth Medium, ready-to-use) for 2 to 8 passages. Both HEK-293 (ATCC) and normal human lung fibroblasts (ATCC) were maintained in DMEM supplemented with 20% fetal bovine serum and antibiotics. For non-micropatterned live-imaging experiments, glass-bottomed imaging dishes were sterilized with deep UV light for 3 min. All cells were maintained in a humidified incubator at 37 °C and 5% CO_2_.

### Immunofluorescence and microscopy

For immunofluorescence, HUVECs were fixed with 4% paraformaldehyde (PFA) for 7 min. ECs were then washed three times with phosphate buffered saline (PBS) and permeabilized with 0.5% Triton-X for 10 min. After permeabilization, cells were washed three times with PBS. ECs were then blocked with 2% bovine serum albumin (BSA) for 10 min. Once blocked, primary antibodies were incubated for approximately 4–24 h. Thereafter, primary antibodies were removed, and the cells were washed 3 times with PBS. Secondary antibodies were added in 2% BSA and incubated for approximately 1–2 h, washed 3 times with PBS, and mounted on a slide for imaging. All primary and secondary antibodies are listed in the Supplemental Information Table 2.

For live imaging, plasmids were used at concentrations between 2 and 3.5 µg per transfection. Plasmids were transfected into HUVECs (Supplementary Table 1) using a ThermoFisher Neon transfection system with a voltage of 1350 V, wavelength of 30 ms, and 1 pulse. Cells were then plated overnight (8–16 h) and transferred to micropatterned coverslips 1–2 h prior to imaging. A TOKAI HIT stage was used for live-cell at 5.0% CO_2_ and 37.0 °C for the duration of the experiment. Images were captured on a Nikon Eclipse Ti inverted microscope equipped with a CSU-X1 Yokogawa spinning disk field scanning confocal system and a Hamamatsu EM-CCD digital camera. Images were captured using a Nikon Plan Apo 60 × NA 1.40 oil objective using Olympus type F immersion oil NA 1.518, Nikon Apo LWD 20X NA 0.95, Nikon Apo LWD 40X NA 1.15, or Nikon Apo WD 10X NA 0.5 objective. All images were processed using ImageJ (FIJI).

### Micropattern fabrication

Except for the Lipidure coating, micropatterning fabrication was carried out as previously outlined by Azioune et al.^4^. Briefly, coverslips were plasma cleaned at atmospheric conditions for 20 s at 25% power. Afterwards, 50 µL of TI Prime (Microchemicals) was spin-coated onto the activated coverslips and then cured on a hot plate at 120 °C for 1 min. Once cured, 0.5% polystyrene dissolved in toluene was spin-coated onto the coverslips. When creating micropatterned glass coverslips, the TI Prime and polystyrene steps were omitted. To adhere the Lipidure to the polystyrene, the coverslips were plasma cleaned again, and then 75 µL of 0.125% Lipidure dissolved in 100% ethanol was spin-coated onto the coverslips. At this point, coverslips can be stored at room temperature. Prior to deep UV exposure, Lipidure-coated coverslips were briefly soaked in PBS for 10–15 min. After rehydration, 35 µL of ddH_2_O was used to adhere the coverslips, Lipidure side down, onto the bottom of a photomask (non-chrome side). The photomask was then placed into a deep UV chamber, chrome side up, for 3 min. Following UV exposure, a liberal amount of water was added to the coverslips to dislodge them from the photomask. Lastly, patterned coverslips were placed on a 35 µL fibronectin (2ug/mL in 100 mM HEPES, pH8) droplet and incubated for 30 min at room temperature to graft fibronectin to the vacated patterned areas. Please see Supplemental Information Table 3 for all reagents associated with fabrication and Supplemental Information Table 4 for a list of required equipment. Also please see the Supplemental Information Extended Procedures for a more detailed step-by-step protocol.

### Line scan and histogram analysis

The lines were drawn perpendicular to the pattern so that one endpoint was at the center of the micropattern and the other was outside of the pattern. Using the PlotProfile function in Fiji (opensource software), data points were copied into Microsoft Excel, and gray values were normalized via relative change from each set’s first data point using the formula x = (I − I_0_)/I_0_ where x is the normalized value, I is the gray value, and I_0_ is the initial gray value. To account for variability in laser power between experiments, outputs were further normalized by multiplying or dividing. Distance (µm) vs. fluorescence intensity plots were then created using GraphPad Prism.

MATLAB was used to make the radial histograms. Nuclear-centrosome angle data was calculated in an excel file that was imported into MATLAB for quantification. Polar axes were set up to accommodate the data. Bins were then determined to be 45 degrees, leaving 8 bins. Then, the plot was loaded with the nuclear-centrosome position data and the number of bins using the built-in MATLAB function ‘polarhistogram.’ Lastly, the histogram was rotated so that zero was on the top of the crossbow pattern using the built-in function ‘ThetaZeroLocation.

### ADAPT quantification

To track actin cellular dynamics, the ADAPT opensource plugin for FIJI ImageJ created by Barry et al.^12^ was employed. The segmented ADAPT distances were imported into GraphPad Prism from the plugin’s output data for graphing.

### Supplementary Information

Supplementary Information.

## Data Availability

Authors will make any data, analytic methods, and study materials available to other researchers upon written request to Dr. Erich J Kushner.
