# Supplementary material for: Lipidure-based micropattern fabrication for stereotyping cell geometry
Source: Sci Rep. 2023 Nov 22;13:20451. doi: 10.1038/s41598-023-47516-8 (PMC10665372; doi:10.1038/s41598-023-47516-8)
Supplement: Supplementary file 1 — Supplementary Information. [file 41598_2023_47516_MOESM1_ESM.pdf]

Supplemental Information: Lipidure-based micropattern fabrication for stereotyping cell geometry

Drew B. Grespin; Talen G. Niven; Riley O. Babson; and Erich J. Kushner*
*Author for correspondence

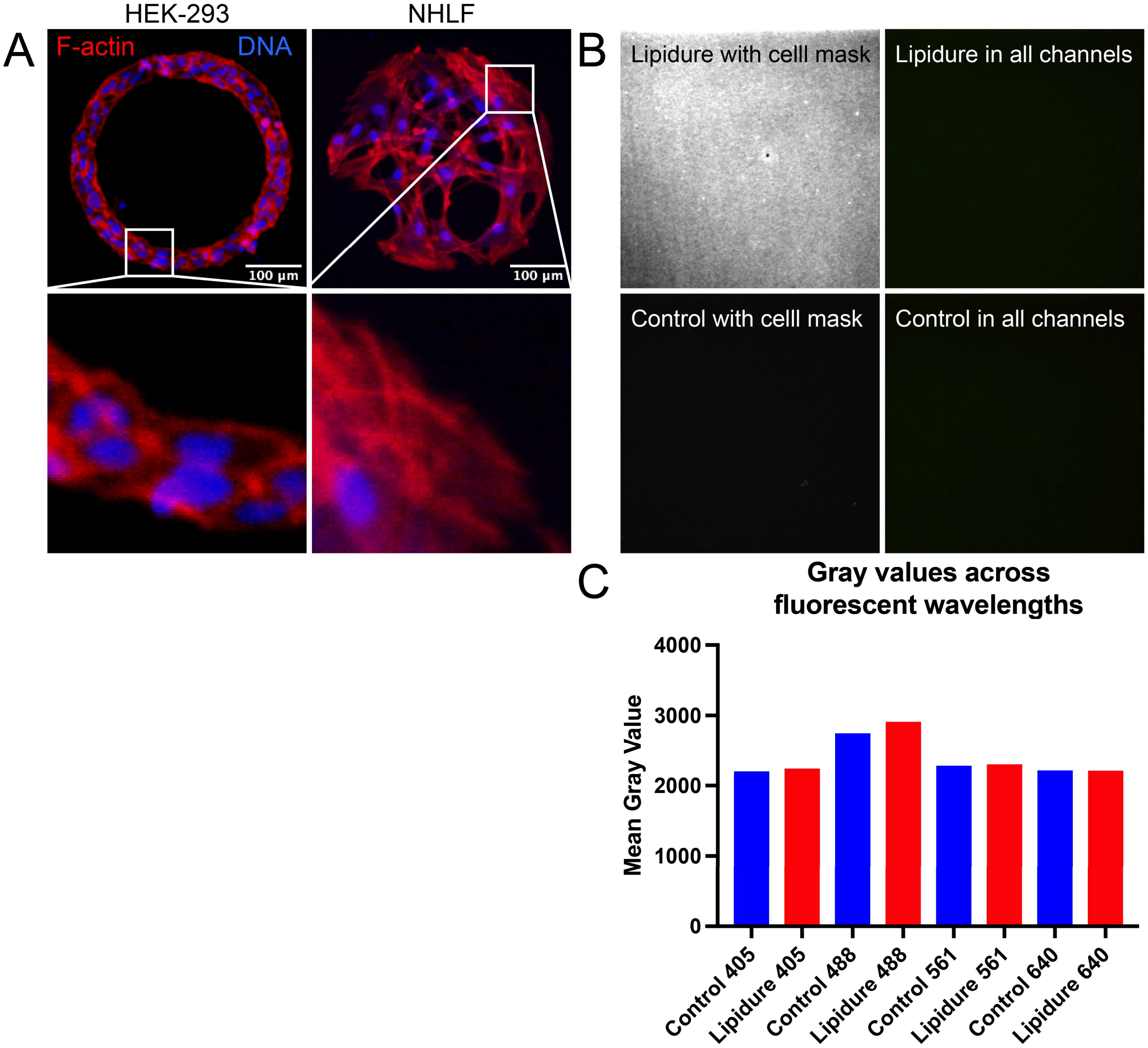

Supplementary Figure 1. Track micropatterns with HEK-293s and NHLFs and transparency tests. (A) Representative images of Human Embryonic Kidney (HEK) 293 cells and normal human lung fibroblasts (NHLFs) adhered to track micropatterns with insets shown below. (B) On the left are representative images taken in the far red (640) channel of Lipidure-coated (top) and non-coated control (bottom) coverslips stained with Cell Mask^{TM}. On the right are representative images taken in the 405nm, 488nm, 561nm, and 640nm channels of Lipidure-coated (top) and non-coated control (bottom) coverslips. (C) Bar chart of mean gray values in the 405, 488, 561, and 640 channels for Lipidure-coated and non-coated control coverslips.

Cost Analysis

Lipidure costs $300USD per 1 gram. PLL(20)-g[3.5]-PEG(2). When purchasing through SuSoS AG, PLL(20)-g[3.5]-PEG(2) costs $12,000 for 1 gram. However, PLL(20)-g[3.5]-PEG(2) can also be purchased through Ruixibio Tech Company and costs $1,550 for 500 milligrams. To determine which substrate is the most cost efficient, we calculated the cost per coverslip. For Lipidure, we assumed 1.25mg/mL Lipidure in EtOH and 75 µL for surface preparation. For both PLL(20)-g[3.5]-PEG(2) prices, we assumed 0.1 mg/mL and 75µL for surface preparation.

- For Lipidure (NOF):
$$\frac{12.5\text{mg}}{10\text{mL}} \times \frac{1\text{g}}{1000\text{mg}} \times \frac{\$300}{1\text{g}} = \frac{\$0.375}{1\text{mL}} \times \frac{1\text{mL}}{1000\mu\text{L}} \times \frac{75\mu\text{L}}{1\text{ Coverslip}} = \$0.028\text{ per Coverslip}$$
- For PLL(20)-g[3.5]-PEG(2) (SuSoS)
$$\frac{0.1\text{mg}}{1\text{mL}} \times \frac{1\text{g}}{1000\text{mg}} \times \frac{\$12{,}000}{1\text{g}} = \frac{\$1.2}{1\text{mL}} \times \frac{1\text{mL}}{1000\mu\text{L}} \times \frac{75\mu\text{L}}{1\text{ Coverslip}} = \$0.090\text{ per Coverslip}$$
- For PLL(20)-g[3.5]-PEG(2) (Ruixibio Tech Company)
$$\frac{0.1\text{mg}}{1\text{mL}} \times \frac{\$1{,}550}{500\text{mg}} = \frac{\$0.31}{1\text{mL}} \times \frac{1\text{mL}}{1000\mu\text{L}} \times \frac{75\mu\text{L}}{1\text{ Coverslip}} = \$0.023\text{ per Coverslip}$$

Based on the cost breakdown, the cost per coverslip of PLL(20)-g[3.5]-PEG(2) purchased through Ruixibio Tech are the least expensive, but the cost per cover slip of Lipidure is still very competitive, only being a difference of 0.05 cents.

EXTENDED PROCEDURES

Detailed Micropatterning Protocol for Lipidure on Glass Coverslip (Main Figure 1a):

Materials:

- 25mm No. 1 glass coverslip
- ddH_2O
- Phosphate-Buffered Saline (PBS)
- 100% Ethanol
- Lipidure (NOF America)
- Trypsin
- Dulbecco's Modified Eagle's Medium (DMEM)
- Phosphate Buffered Saline (PBS)
- Endothelial Growth Media (EGM)
- Deep Red Plasma Membrane Stain (Thermo Fisher Scientific)

Equipment:

- Plasma cleaner
- Deep-UV chamber
- Spin coater
- Transparency chrome photomask
- Incubator

Lipidure on Glass Surface Preparation:

1. Retrieve 25 mm coverslips and place in a plasma cleaner using specified running conditions (Plasma clean: 20 s; Vacuum set point: 200.3; Atmospheric vent: 30 s; Purge Vent: 10 s; Gas Stabilize: 20 s; Vacuum alarm: 3:00 min; Auto cycle off: On; Plasma Power: 20%)
2. Once cleaned, place coverslips onto a spin coater centered on chuck and apply vacuum.
3. Run the spin coater for 30 seconds (s) at 2000 RPM.
4. After reaching 2000 RPM, pipette 75 µL of 0.125% Lipidure dissolved in 100% ethanol dropwise onto the center of the rotating coverslip.
5. After run completion, disengage the vacuum, and remove the coverslip using forceps, grasping the edge to avoid scratching the Lipidure-coated surface.
6. After this step, the Lipidure-coated slip is stable for at least 90 days.
7. Place the coated-coverslips into a 35 mm dish face-up and submerge in PBS for at least 15 minutes (min) to hydrate the Lipidure prior to masking.

Masking:

1. Thoroughly clean the photomask with ethanol/acetone and/or soap and H_2O until it is streak free.
2. Apply 50 µL of ddH_2O in the center of the pattern on the bottom of the photomask (non-chrome side).
3. With forceps, obtain a freshly hydrated Lipidure-coverslip. Hold the coverslip vertically and blot the excess PBS on a Kimwipe.

4. Place the coverslip on the photomask Lipidure side down.
5. Using a folded Kimwipe gently apply pressure to the coverslip, which will force excess H_2O from under the coverslip and ensure strong adherence to the photomask. The Kimwipe will absorb the expelled liquid.
6. Once the coverslip is adhered to the photomask, turn the photomask over (chrome side up), and place it into a deep-UV chamber roughly 7 cm away from the lamp.
7. Expose the photomask in the deep-UV chamber for 3 min.
8. Remove the photomask and coverslips from the deep-UV chamber. To remove coverslips apply a generous amount of H_2O around the coverslips. The coverslip will eventually dislodge.
9. Using plastic tweezers or a micropipette tip, nudge the coverslips to the edge of the photomask and remove with forceps.
10. Place coverslips Lipidure side up back into a clean 35 mm dish.
11. Wash the slips 3x with sterile PBS and proceed to cell deposition.
12. If sterility is desired, see Heat Sterilization protocol below.
13. OPTIONAL: Immerse coverslips in 2 mL of PBS containing 2 µL of Deep Red Plasma Membrane Stain and cover with aluminum foil for 10 min. Remove dye solution (this can be collected and used again) and wash 3x with PBS. Coverslip can now be imaged to evaluate the micropatterned features. Periodically, checking for proper feature resolution after photomasking will ensure the process was carried out correctly prior to plating cells.

Heat Sterilization:

1. Remove the patterned coverslip from the 35 mm dish and place it face up to dry on a Kimwipe. Be careful not to touch the patterned surface while drying.
2. Remove as much PBS from the dish as possible using a transfer pipette.
3. Use a Kimwipe to dry the inside of the dish thoroughly.
4. Place the coverslip(s) into a sterile petri dish.
5. Place the dish into an oven at 100ºC for 10 min to sterilize the coverslips.
6. Remove the dish from the oven for storage or for cell seeding after cooling back to room temperature.

Endothelial Cell Deposition:

1. Inside a Biosafety cabinet, remove media from a HUVEC culture and wash 3x with PBS. Remove PBS and apply 1 mL of trypsin-EDTA, return the plate to the incubator for 2 minutes.
2. Once cells are detached, neutralize Trypsin with 5 mL DMEM containing 10% fetal bovine serum and antibiotics.
3. Centrifuge the resulting cell suspension at 500xg for 4 min.
4. Aspirate the supernatant off.
5. Resuspend the cells in complete EGM using 2 mL of EGM for every coverslip that will be plated.
6. Return the cells to the incubator for 20-30 min if using fibronectin, 1-2 hours if plating on glass, to allow for minimal adherence to the patterned areas.
7. Once cells begin to adhere, remove the cell suspension (this can be saved) and wash with PBS to remove non-attached cells.
8. After washing, replace the EGM for each coverslip.
9. Depending on the cell type and desired patterns, incubate the micropatterned cells at 37ºC for 3 hours before staining, fixing, and/or imaging.

Lipidure on Polystyrene Surface Preparation:

1. Retrieve 25 mm coverslips and place in a plasma cleaner using specified running conditions.[1]
2. Place the coverslip onto a spin coater centered on chuck and apply vacuum.
3. Apply a vacuum to the slip being spin coated.
4. Run the spin coater for 30 s at 3000 RPM.
5. Immediately after reaching 3000 RPM, pipette 50 µL of TI Prime onto the center of the coverslip.
6. Allow the spin coater to complete its cycle prior to disengaging the vacuum.
8. Disengage vacuum and remove the coverslip using forceps and grasping the edge to avoid scratching the TI Prime-coated surface.
7. Cure the TI Prime at 120ºC for 1 min on a hotplate.
8. Repeat steps 2-4.
9. Immediately after reaching 3000 RPM, use a glass Pasteur pipette to place 4 drops of 0.5% polystyrene dissolved in toluene onto the center of the rotating coverslip.
10. Repeat step 1 (plasma cleaning).
11. Lipidure is added as stated in *Lipidure on Glass Surface Preparation* section, beginning at step 2. All other procedures are the same as stated in above sections.

TABLE 1: PLASMIDS

Type	Name	Concentration	Addgene number
Plasmid	pLL 7.0*	2000ng/transfection	In house
Plasmid	pLentiCMV-LifeActTagRFP647p2aCentrin-GFPt2aNLSmCherry	2000ng/transfection	In house

TABLE 2: ANTIBODIES AND DYES

Type	Name	Vendor	Concentration	Catalog Number
Primary Antibody	Gamma Tubulin	Thermo Scientific	1:1000	MA1-20248
Primary Antibody	Rabbit polyclonal to VE cadherin – intercellular junction marker	Abcam	1:1000	Ab33168
Secondary Antibody	Goat anti-Rabbit IgG (H+L) Highly Cross-Adsorbed Secondary Antibody, Alexa Fluor Plus 488	Thermo Scientific	1:1000	A32731
Secondary Antibody	Goat anti-Mouse IgG (H+L) Highly Cross-Adsorbed Secondary Antibody, Alexa Fluor Plus 488	Thermo Scientific	1:1000	A11001
Conjugated Stain	Alexa Fluor 555 Phalloidin	Thermo Scientific	1:500	A34055
Conjugated Stain	Alexa Fluor 647 Phalloidin	Thermo Scientific	1:500	A22287
Stain	Hoechst 33342 Trihydrochloride, Trihydrate – 10 mg/mL solution in water	Thermo Scientific	1:1000	H3570
Stain	Cell Mask™ Plasma Membrane Far Red Stain	Thermo Scientific	5mg/mL	C10046

TABLE 3: MATERIALS

Material	Vendor	Catalog Number
Toluene	Sigma-Aldrich	244511-1L
Polystyrene pellets	Sigma-Aldrich	182436-25G
Lipidure-CM5206	NOF America Corp.	CM5206

TI Prime	MicroChemicals	
Fibronectin bovine plasma	Sigma-Aldrich	F4759
Recombinant Human VEGF 165 Protein, CF	R&D	293-VE-010
SCH772984	Selleckchem	S7101
GenClone Dulbecco's PBS without Calcium, Magnesium	Genesee Scientific	25-508B
Endothelial Cell Growth Kit-VEGF	ATCC	PCS-100-041
Vascular Cell Basal Media	ATCC	PCS-100-030
Penicillin-Streptomycin 100X Solution	Genesee Scientific	P4333-100ML
Paraformaldahyde 20% Aqueous Sol. EM Grade	Electron Microscopy Sciences	15713
DMEM, High Glucose, with LGlutamine	Genesee Scientific	25-500
Trypsin-EDTA, 0.25% 1X, phenol red	Genesee Scientific	25-510
GenClone Tissue Culture Dishes (10 cm)	Genesee Scientific	25-202
25 mm Cover Glass No. 1 Thickness	Thor Labs	0111650
GenClone Tissue Culture Plate 6-well, Flat Bottom	Genesee Scientific	25-105
Bovine Serum Albumin, Fraction V, Purity >98%, pH 7.0	Prometheus	25-529

TABLE 4: EQUIPMENT

**Device**	**Company**
Photomask	Photomask Portal
Spin Coater Model WS-650Mz-23NPPB	Laurell Technologies
Plasma Cleaner PE-25LF	Plasma Etch
Jelight Deep-UV Model 24	Jellight Company, Inc.
Nikon Eclipse Ti inverted microscope	Nikon
Laminar Flow hood 1300 Series A2	Thermo Scientific
CO_2 Incubator MCO-170AICUVDL	Phcbi
Neon® transfection system	Thermo Scientific
